# Thermoset Polymer Matrix Structure and Properties: Coarse-Grained Simulations

**DOI:** 10.3390/polym11010036

**Published:** 2018-12-27

**Authors:** Vladimir Yu. Rudyak, Elizaveta A. Efimova, Daria V. Guseva, Alexander V. Chertovich

**Affiliations:** Faculty of Physics, Lomonosov Moscow State University, Leninskie gory, 1-2, Moscow 119991, Russia; elizaveta.a.efimova@gmail.com (E.A.E.); guseva@polly.phys.msu.ru (D.V.G.); chertov@polly.phys.msu.ru (A.V.C.)

**Keywords:** polymers, networks, phthalonitrile, curing, simulations, mesoscale chemistry, dissipative particle dynamics, elastic modulus, network topology

## Abstract

The formation of a thermoset polymer network is a complex process with great variability. In this study, we used dissipative particle dynamics and graph theory tools to investigate the curing process and network topology of a phthalonitrile thermoset to reveal the influence of initiator and plasticizer concentration on its properties. We also propose a novel way to characterize the network topology on the basis of two independent characteristics: simple cycle length (which is mainly affected by the initiator amount) and the number of simple cycles passing through a single covalent bond (which is determined primarily by plasticizer concentration). These values can be treated in the more familiar terms of network “mesh size” and “sponginess”, correspondingly. The combination of these two topological parameters allows one to characterize any given network in an implicit but precise way and predict the resulting network properties, including the mechanical modulus. We believe that the same approach could be useful for other polymer networks as well, including rubbers and gels.

## 1. Introduction

Polymer composite materials, or fiber-reinforced plastics, are rapidly being introduced into many areas of our lives and dramatically changing all constructional material industries [1]. The demands placed on filler materials are more-or-less understandable (they should be lightweight and strong, such as carbon fiber). At the same time, the properties of polymer matrices are much more sophisticated, and their influence is often underestimated. Nevertheless, matrix mechanical properties and their coupling with filler stiffness (which is usually hard to change) primarily determine the overall composite properties for the consumer, including static and dynamic moduli, elastic and brittle behavior, etc. Moreover, on the practical side, it is much easier to tune matrices than it is the filler, because the filler is mostly “as is” material produced by very few manufacturers on the basis of closed technologies [2]. The other two important contributors to composite material properties are the filler arrangement inside the matrix and filler–matrix interface properties, which are also very sophisticated and widely studied nowadays, but these are out of the scope of our research.

**Figure 1:**
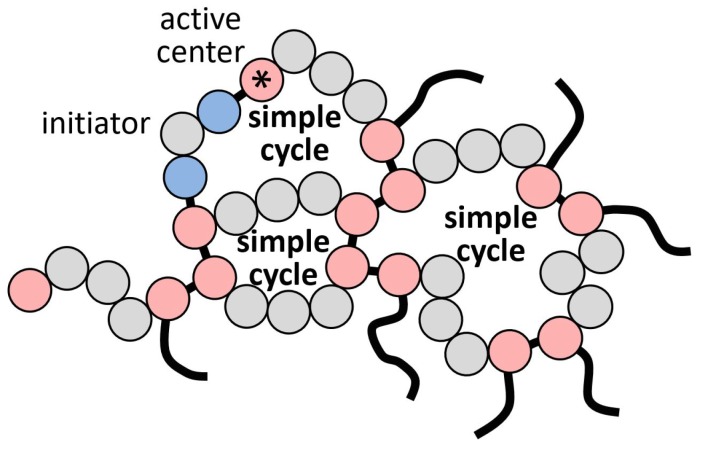
Phthalonitrile network during the curing process.

The polymer matrix itself is a percolated three-dimensional network of monomer units connected by covalent bonds, as shown in Figure 1. These bonds are formed during a chemical polymerization reaction, usually called the curing process. In addition to monomer units, some other species can be added, like hardeners and plasticizers, to control the reaction speed and crosslink density. Depending on the monomer’s chemical structure, the nature of the polymerization reaction can be either a step-growth process (as in the crosslinking of phenolic or epoxy resins) or radical polymerization (as in the crosslinking of polyester resin or acrylic glass). The latter case requires an initiator to start the reaction and usually takes more time. In addition, radical polymerization leads to a more complex topology of the network and contains defects, like dangling ends. In this study, we consider only the case of radical polymerization, although our results are probably also applicable to step-growth polymerization, reduced by the effects of initiator concentration.

In general, there is a vast number of different chemical components which can be polymerized or copolymerized to form a resin. While the chemical nature of the monomers is very important for the details of the curing process and also controls the glass transition temperature and heat resistance, the mechanical properties depend strongly on the topology of the final polymer network. The network topology, in turn, depends on many factors (such as species composition, reaction pathways, and conversion degree) and, in most cases, is not known both a priori and a posteriori.

The influence of the polymer network topology is a well-known problem for polymer elastomers, including rubbers and especially gels [3,4]. Usually, the real network topology in elastomers is unknown, except for in cases in which the “network disassembly spectrometry” technique can be applied [5]. In these cases, a straightforward comparison between the chemical topology and the theoretical model can be done [6,7]. However, this approach does not work for resins; thus, only a few attempts to study resin network topology are found in the recent literature [8,9]. The variation in polymer network crosslinker functionality and its influence on topology and mechanical properties was studied for the two-dimensional case in [10]. The study of gel point formation for a universal polymer network model was presented recently [11]. This model is able to account for loop formation, which is most important for curing in a swollen gel state. The closest to our work is found in [12], wherein the authors used simulations to s study the radical polymerization of hexanediol diacrylate with varied initiator content. Here, we studied the network topology at a more detailed level and combined both the effects of low-molecular-weight plasticizer and initiator concentration.

A common and very approximate viewpoint is that, on average, a shorter distance between crosslinks (usually called “mesh size”) yields a stiffer and brittler matrix, while larger distances result in softer and more elastic materials. In this sense, it correlates with the classical polymer rubber properties, and the correspondence between soft resin (in the region above the glass transition temperature) and highly crosslinked rubber is obvious.

The phthalonitrile resin is one recent example of various modern resins [13,14], and it is thermally much more stable than the classic epoxy resin. It polymerizes via a complex radical process and has a complex network topology. These resins are more expensive compared with the epoxy ones but can be used to create composites with a high temperature load for such uses as turbine blades or hyper-velocity aerodynamic shrouds. With the radical polymerization process, the initiator species should be introduced to start the curing, and the initiator concentration is varied to control the curing time and to increase the final conversion. At the same time, a plasticizer can be used as an inert additive to affect the elastic modulus and fragility.

In parallel to the development of novel chemical species, computer simulation in silico experiments are attracting attention for studying and predicting various material properties, both at the atomic level and the coarse-grained level [15]. In our recent works, we developed a multiscale simulation methodology that makes it possible to predict various physical properties of highly crosslinked polymer materials [16,17,18], but there is still a lack of understanding such a network topology and the possible connections of the network topology with macroscopic properties.

Taking in mind the polymer matrix properties, let us emphasize once more that the polymer network topology is formed during the curing process, which is usually fully controlled by the final product makers. Thus, the main purpose of our research is to build a computer model and describe the influence of the curing process input parameters on the final polymer matrix topology and mechanical properties. As the input parameters, we used the initiator and plasticizer concentration, which determine the matrix network topology, followed by the corresponding mechanical properties. Because of the substantial polymer resin diversity, we considered the specific example of the phthalonitrile resin, as it is one of the most interesting and promising cases. However, the same conclusions are most likely valid for any thermoset resin.

**Figure 2:**
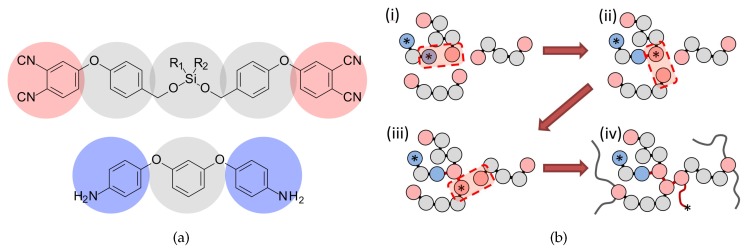
Representation of the coarse-grained model. (**a**) Coarse-grained mapping schemes of phthalonitrile monomer p-SiMe${}_{2}$PN and initiator (diamine curing agent 1,3-*bis*(4-aminophenoxy) benzene). (**b**) The scheme of the “mesoscale chemistry” reactions of initiation (i–ii) and polymerization (ii–iv). Terminal beads of each monomer (red) and initiator (blue) have valences of 2 and 1, respectively; asterisk indicates an active bead. Red arrows show reaction pathways.

Please note also that, in our model, there are no real defects, such as dangling ends or large dangling loops. This is because of the monomer and initiator units’ special design (see Figure 2b), which prohibits the formation of such defects. This means that we study defect-free networks and can measure the influence of network topology solely, disregarding any dangling or other elastically inactive elements.

## 2. Methodology

We produced computer simulations of matrix curing, equilibrated the resulting networks, and studied their topology and mechanical properties using dissipative particle dynamics (DPD) simulations. DPD is a mesoscopic simulation method that was originally proposed by Hoogerbrugge and Koelman [19,20] and later developed for the simulation of polymers and molecular systems by Espanol, Groot, and Warren. In this method, a bead-and-spring model is used to represent molecules, and beads move according to Newton’s equations of motion and interact with conservative (repulsion), dissipative (friction), and random (heat generator) forces. The use of soft repulsive potential increases the numerical stability of the integration of the equations of motion, which makes it possible to achieve a higher integration time step (compared with molecular dynamics or Brownian dynamics) and, consequently, to simulate complex polymer networks on larger time and spatial scales. Recently, the “mesoscale chemistry” concept was successfully adopted using this method for simulations of polymerization reactions [8,16,21].

### 2.1. Coarse-Grained Model

In this study, we considered polymer networks based on the phthalonitrile monomer p-SiMe${}_{2}$PN and, as the initiator, the diamine curing agent 1,3-*bis*(4-aminophenoxy)benzene; thus, this work is a continuation of our recent investigations of such systems [16,17,18]. The coarse-grained representation of initial chemical structures is shown in Figure 2. We assumed that all beads had almost the same volume and the same corresponding DPD pair repulsion parameter a=90 (details of the choice can be found in [17]) at temperature T=1 and number density ρ=3. Harmonic bond (equilibrium bond length $l_{b}=0.5$, bond strength $K_{b}=90$) and angle potentials (equilibrium angle $\gamma_{a}=180^{\circ }$, angle strength $K_{a}=1.8$) were applied, which required using a time step of $\delta t=5\times 10^{-3}$ for the stable performance of the integration scheme. The initial simulation box had a size of 30×30×30 DPD units, which is approximately 23×23×23 nm${}^{3}$ at room temperature, according to known density data [22]. We prepared eight systems with different ratios of the initial compounds. Four systems contained only monomer and initiator, with mass proportions of 99:1 (16,038 monomers), 96:4, 92:8, and 84:16 (13,608 monomers). Another four systems contained monomer, initiator, and low-molecular plasticizer. In these systems, the monomer-to-initiator mass ratio was fixed at 96:4, and the plasticizer mass varied from 8% to 64% (5598 monomers) of the total mass of the system. Terminal beads of monomer and initiator molecules (shown in red and blue in Figure 2) had free valences equal to 2 or 1, respectively, and could be either inactive (initially for monomers) or active (initially for initiator molecules).

### 2.2. Curing Process Simulation

In this work, the propagation process in radical polymerization reactions was simulated as follows. Active beads were able to form bonds with inactive terminal beads and passed an active state to the bead with which they formed a bond. This model involves the concept of “mesoscale chemistry” and was described in detail in our recent papers [16,17]. We implemented two types of reactions at the mesoscale level:

1. Initiation reaction, during which terminal beads of initiator and monomer molecules form a bond, and the initiator terminal bead passes the active state to the corresponding monomer bead (asterisk in Figure 2b(i–ii).
2. Polymerization reaction, during which an active bead of the monomer forms a bond with a neighboring terminal bead and passes the active state to that bead, as in Figure 2b(ii–iii). This process repeats until the conversion rate reaches 100%, as depicted in Figure 2b(iii–iv).

Both reactions were carried out in the NVT ensemble. At regular time intervals (10 DPD time units, or 2000 DPD steps), each pair of active terminal bead and neighboring terminal bead having free valences was tested for the following condition: if the distance between these beads was less than the reaction radius $R_{c}=1$, then a new bond was formed between these beads with the probability p=0.01. The time interval and reaction probability were chosen to be sufficiently small to maintain quasi-equilibrium conditions [21].

During the curing process simulations, two parameters were evaluated: the conversion degree *c* and the fraction of particles belonging to the largest cluster in the system $m_{c}$. The conversion degree was calculated as the ratio between the number of bonds created and the maximum possible number of new bonds. The fraction of the largest cluster was determined as the ratio between the largest cluster size and the total number of monomer and initiator beads in the system (i.e., the whole system excluding plasticizer).

### 2.3. Calculating Matrix Properties

We studied the mechanical properties of coarse-grained matrices. For each of eight prepared systems, a matrix with a conversion degree of c=95% was analyzed. We applied a uniaxial deformation along the *x*-axis to the simulation box and measured stress–strain response curves. During deformation, the volume of the box was kept constant. Deformation was applied at a slow enough rate to keep the system equilibrated during simulations ($\lambda =L/L_{0}=1.66$ was reached in $10^{5}$ DPD time units). The components of the stress tensor *p* were averaged using the virial theorem [23]. The true stress was calculated as $\sigma =\langle p_{xx}\rangle -0.5\langle p_{yy}+p_{zz}\rangle$.

Then, we used two models to estimate the elastic modulus *E*: the Flory model and the Three Chain Model. The Flory model [24] considers systems of densely packed free joint networks with segment length *l* and contour length *L* to be affinely deformed. Let the deformations of primary axes *x*, *y*, and *z* be $\lambda_{x}$, $\lambda_{y}$, and $\lambda_{z}$, correspondingly. Considering affine deformations of each subchain, one can express the change in the free energy of a single subchain as the following:

$$\delta f=\frac{3k_{B}T}{2Ll}\left(R^{2}-R_{0}^{2}\right)=\frac{3k_{B}T}{2Ll}\left(R_{x0}^{2}\left(\lambda_{x}^{2}-1\right)+R_{y0}^{2}\left(\lambda_{y}^{2}-1\right)+R_{z0}^{2}\left(\lambda_{z}^{2}-1\right)\right).$$

It was shown [25] that, for the case of uniaxial deformations with a constant sample volume ($\lambda_{x}=\lambda$, $\lambda_{y}=\lambda_{z}=\lambda^{-0.5}$), the stress can be then written as follows:

$$\sigma =\frac{1}{V}\frac{dF}{d\lambda }=k_{B}T\nu \left(\lambda -\frac{1}{\lambda^{2}}\right),$$

where ν is a number of subchains per unit volume. This equation leads to a sufficiently nonlinear stress–strain dependency, which coincides with known experimental and simulation data for small deformations.

However, larger elongations require a more complex approach due to the entanglements between the subchains and finite extensibility of subchains. The Three Chain Model (TCM) [26] takes into consideration the non-Gaussian behavior of subchains under deformations. It considers a system as an ideal network formed by cells with three separate subchains in each cell (by the number of dimensions). Considering affine deformations of all subchains, one can express the stress σ via an inverted Langevin function $L^{-1}$:

$$\sigma =\frac{\nu k_{B}T}{3}n^{1/2}\left\{L^{-1}\left(\lambda \cdot n^{-1/2}\right)-\lambda^{-3/2}L^{-1}\left({\left[\lambda \cdot n\right]}^{-1/2}\right)\right\},$$

where *n* is a subchain length, and ν is the same value as in the Flory model.

The simulated stress–strain curves were fitted by both models; then, the elastic modulus *E* was considered numerically equal to ν, as supposed in these models.

## 3. Results and Discussions

### 3.1. Curing Speed and Gelation Point

**Figure 3:**
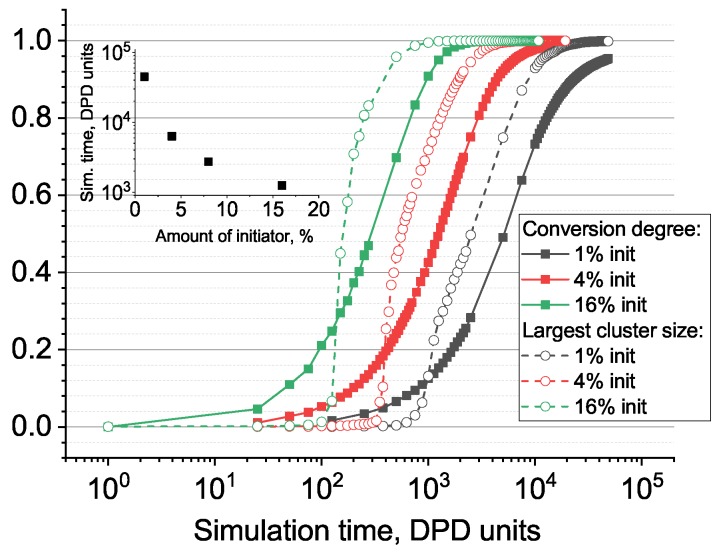
Conversion degree and fraction of particles belonging to the largest cluster as functions of the simulation time. The inset shows the simulation time required to obtain 95% conversion degree depends on the amount of the initiator.

First, let us consider the positive effect of the initiator additive to the overall processing time. Indeed, the processing time itself is a very important feature in any industry-related system, because the time needed to leave a fabric unit in a mold form is critical to the entire manufacturing workflow. Figure 3 gives an overall view of the influence of the initiator concentration on the curing time. We also present the simulation time to reach a 95% conversion degree versus the initiator amount in the inset.

**Figure 4:**
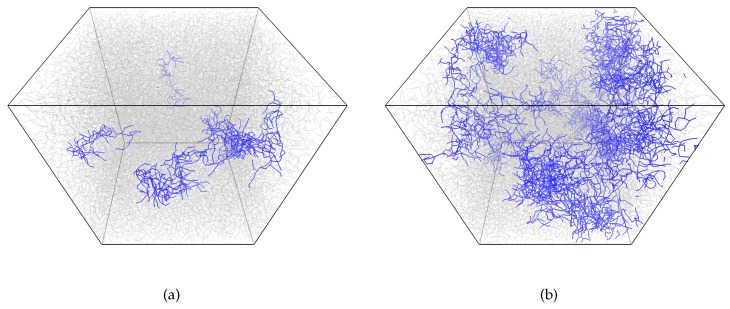
Snapshots of systems with 4% initiator (**a**) before (conversion degree c=0.16, largest cluster size $m_{c}=0.02$) and (**b**) after (c=0.18, $m_{c}=0.11$) gelation point. The largest clusters are marked in blue for both systems.

**Figure 5:**
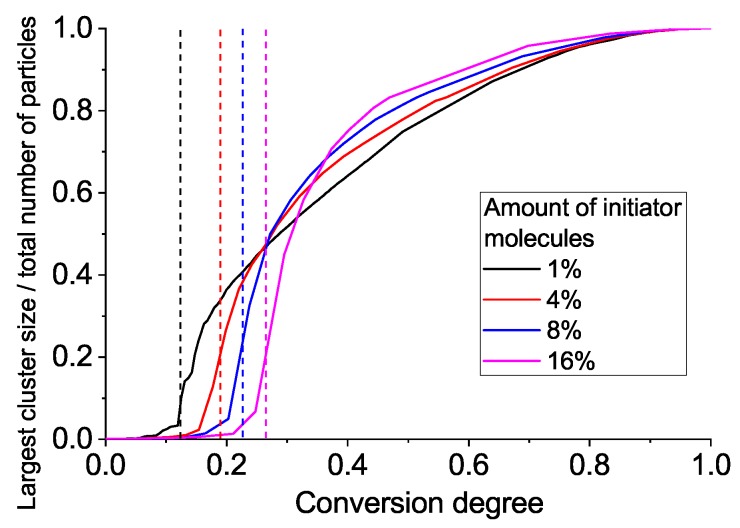
The largest cluster size versus conversion degree for various amounts of initiator.

**Figure 6:**
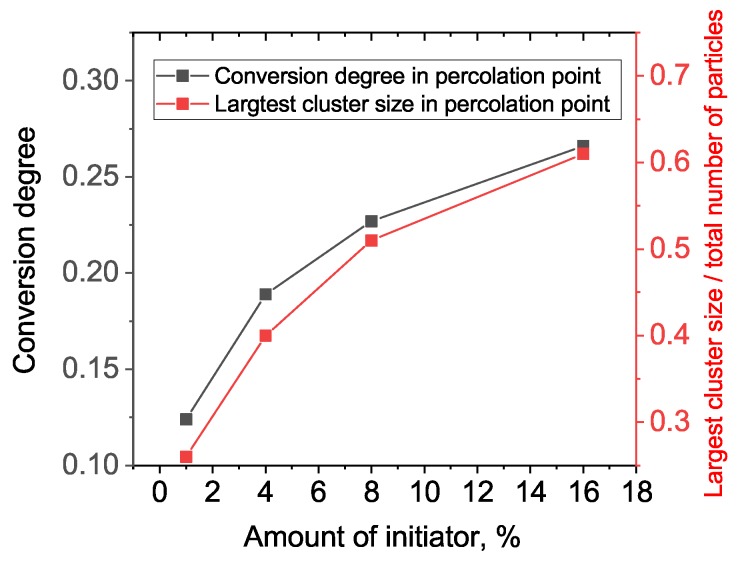
Averaged network properties at percolation point for various amounts of initiator.

Let us discuss, in more detail, the process of gelation and which properties of the network form near the sol–gel transition (i.e., percolation threshold). Figure 4 gives snapshots of the network structure below and above the gelation point, while Figure 5 gives the largest cluster size versus conversion plot. We observe a very heterogeneous network with several growing clumps crosslinked with each other upon gelation. Such heterogeneity is, of course, the consequence of each monomer unit’s valence being equal to four, which leads to the formation of a very dense crosslinking network. An interesting conclusion can be made from Figure 5: the system with a higher amount of initiator has a more delayed and sharp gelation transition (note: here, we talk in terms of conversion along the abscissa axis, not real time). This results in an increased conversion degree and the largest cluster size at gel point, as can be seen from Figure 6.

Thus, we have one more important effect of the initiator, in addition to the enhancement of overall reaction speed: the more initiator we have, the greater the fraction of monomer units included in the gel fraction just after the gel point. That is, at 1% of initiator, there is only 25% of all units included in the first percolation cluster, but if we use 16% of initiator, the percolation cluster contains more than 60% of all monomers. So, we propose that a sample with an increased percolation cluster size can be removed from the molding just after the gelation point, while a sample with a smaller percolation cluster should be exposed to additional post-gelation curing to avoid deformation and leakage after molded form removal.

We also tested the percolation cluster properties in the systems with a varied amount of plasticizer, but there was no significant influence of the plasticizer on the percolation threshold or maximum cluster size, so we do not present these results here.

### 3.2. Mechanical Properties

**Figure 7:**
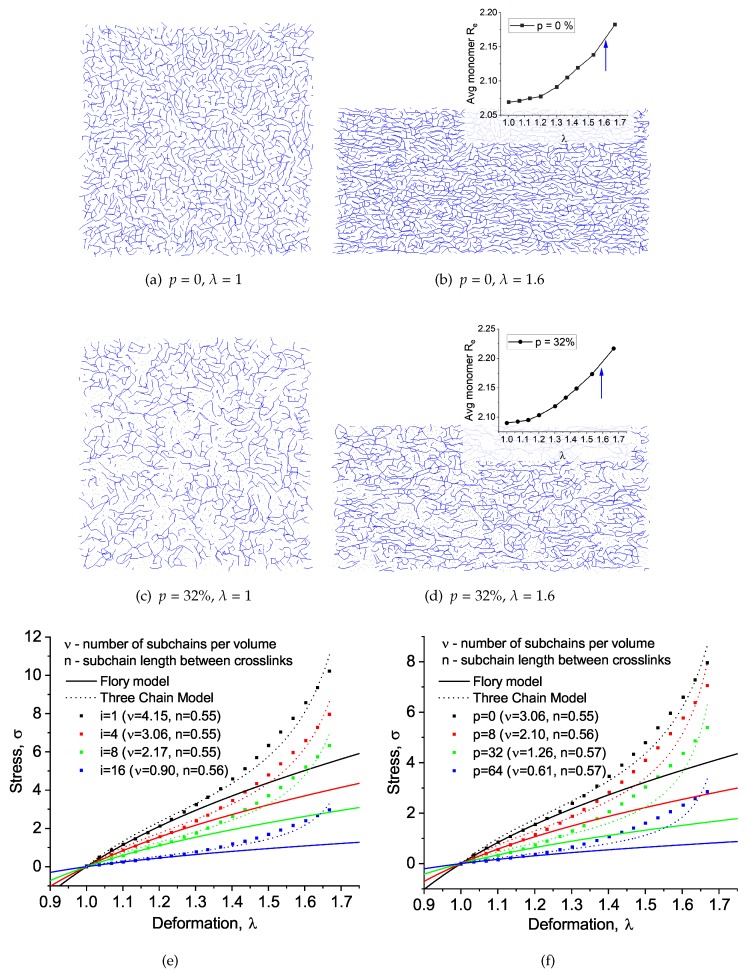
Snapshots of systems with 0% plasticizer and 32% plasticizer at non-deformed (λ=1) and after deformation (λ=1.6) (**a**–**d**). Insets show the average monomer end-to-end distance $R_{e}$ as a function of λ. Stress–strain curves for various initiator concentration of matrices with various amounts of (**e**) initiator and (**f**) plasticizer.

We performed network uniaxial constant volume deformations to study the system’s mechanical properties, as shown in Figure 7 (here, *i* and *p* refer to the amount of initiator and plasticizer, correspondingly). In the upper part of Figure 7, the 2D slice snapshots are presented in equilibrium and deformed state (λ=1.6), while, below, the stress–strain curves are shown. One can see slight alignment along the elongation axis in the deformed state. In addition, there is sufficiently great monomer stretching at λ>1.3−1.4 (see averaged monomer end-to-end distance in insets in Figure 7). This is likely responsible for the rapid nonlinear increase in stress during high elongation, which is not predicted by the Flory model. We also note that the non-uniform direction of these fully stretched segments, especially for the system with the plasticizer, shows that the deformation of the internal network structure is non-affine.

We are well aware of the entropic nature of the elastic deformations of amorphous (non-glassy) networks, while most thermoset systems in the working temperature interval are in the glassy state. Nevertheless, such analysis could be useful to compare with standard polymer elastomer models and to give some estimate of general network properties. The comparison with the polymer elasticity model was made via the simpler Flory affine network model [27] for a region of small deformations 1.0<λ<1.2 (i) and later with the more sophisticated Three Chain Model over all λ values and taking into account the crosslink volume density (ν) from the Flory model. The detailed fitting formulas are given in the Methodology section.

To summarize, the standard Flory model works nicely for small deformations, and the ν value gives the exact measure of the elastic modulus (in kT units). For the greater deformations, the TCM fit overestimates the nonlinear increase in stress caused by the subchains’ final expansibility, but the fitting parameter *n*, which is proportional to the average mesh size, is always close to the same value, which is around 0.56. This overestimation most likely comes from the irregularity of our modeled network: there are no equally distributed cubic meshes inside the network topology, which is assumed for the TCM.

### 3.3. Topology of the Cured Networks

**Figure 8:**
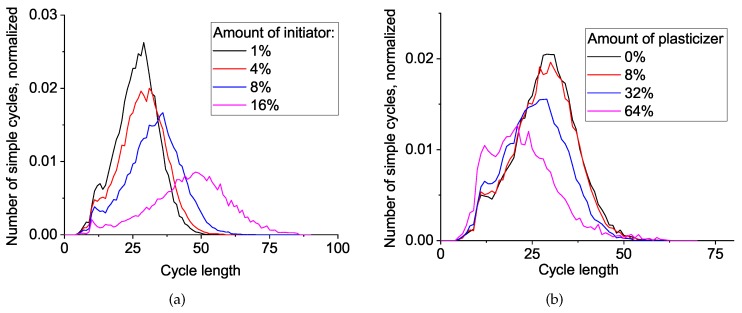
Distribution of simple cycle lengths in matrices with various amount of (**a**) initiator and (**b**) plasticizer. The number of simple cycles was normalized by dividing it by the number of particles in the polymer matrix.

To understand the differences in network topology for systems with various initiator and plasticizer concentrations and to correlate these differences with mechanical properties we studied simple cycles distribution (see Figure 8). In all cases, the cycles are distributed around a Gaussian bell shape, so we can address the average cycle length value and its distribution width (i.e., dispersity). We can clearly observe an increase in mean cycle length and dispersity upon the increase of initiator concentration. This trend is more or less obvious and can be explained by the inclusion of the valuable fraction of a two-valence initiator into the melt of four-valence monomers.

The more intriguing result comes from the plasticizer influence: there is a noticeable decrease in the mean cycle length upon increasing the plasticizer amount. To some extent, this is caused by the redistribution between the standard network mesh size and self-loops from two neighboring monomer units because of the plasticizer-driven dilution effect of active ends, similar to that observed in [11]. However, the other contribution is not obvious and should be discussed in more accurate terms.

**Figure 9:**
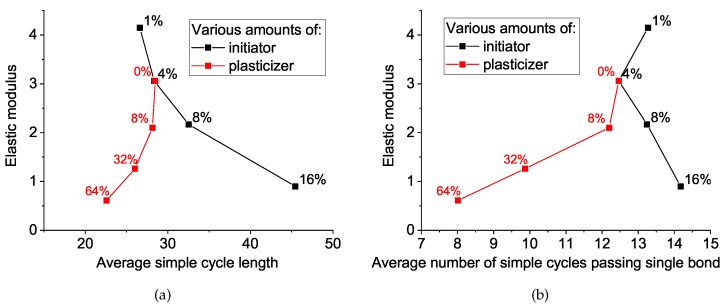
Dependencies of elastic modulus of matrices with various amount of initiator and plasticizer on (**a**) the average simple cycle length and (**b**) the average number of cycles passing through a bond.

To do this, we calculated one more network property: the average number of cycles passing through the bond, which we correlated with the elastic modulus, together with the average cycle length, as seen in Figure 9. The elastic modulus decreases during the increase in initiator concentration (black curve) in straightforward accordance with the simple cycle behavior. However, the situation with the plasticizer concentration is the opposite and, at first glance, counterintuitive: the decrease in cycle length yields a decrease in the elastic modulus. The preliminary conclusion here is the following: both additives (initiator and plasticizer) affect the network properties and considerably decrease the elastic modulus, but the mechanisms are transversal. In the next session, we try to rationalize the aforementioned difference in the mechanism of network softening by the initiator and plasticizer.

Additionally, we calculated the conformational properties of the cycles, namely, spatial distance $R_{ij}$ versus distance along the chain n=i−j, for any pair *i* and *j* inside a simple cycle. However, these plots were almost the same for all cases, indicating Gaussian conformation with $R=n^{1/2}$; we do not present the corresponding plots here.

## 4. Conclusions and Outlook

In this paper, we study the thermoset polymer matrix and the influence of plasticizer and initiator on the gelation process and final matrix properties. The gelation process depends primarily on the initiator amount, and gelation time is inversely proportional to the initiator concentration. In addition, we found that the average gel fraction at the gel point is much higher for the case of a higher initiator amount, which could be of practical interest for potential industry workflows. However, the flip side of the coin is that the initiator increases the mesh size and network softness. The plasticizer does not speed up the curing time, but it also increases network softness.

In addition, in this paper, we propose to calculate one new network topology characteristic, namely, the number of simple cycles passing through a single bond. To the best of our knowledge, this the first time this characteristic of network topology is considered in addition to cycle distribution, which is often studied in state-of-the-art papers on polymer networks [9,12].

**Figure 10:**
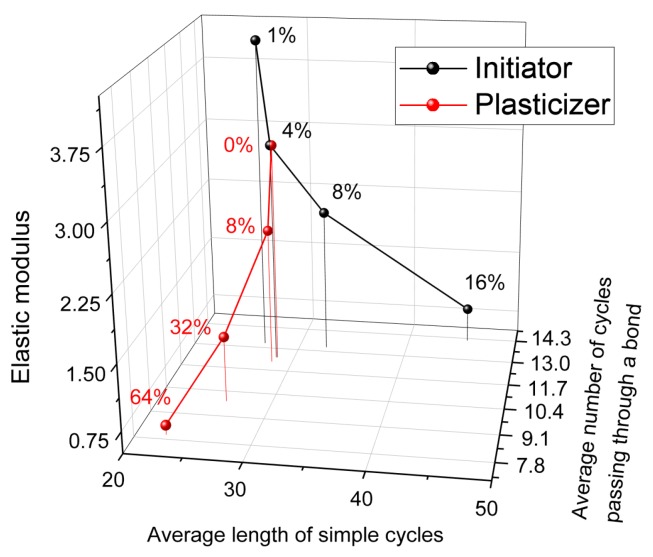
Elastic modulus as a function of average simple cycle length and average number of cycles passing through a bond for systems with varying amounts of initiator (black) and plasticizer (red).

We correlated mechanical and topological network properties and propose a valuable way to characterize network softness based on two topological properties: average simple cycle length and average number of simple cycles passing through a single covalent bond. These values can be treated in the more familiar (although inaccurate) terms of network “mesh size” and “sponginess”, correspondingly. We found that, in the studied case of phthalonitrile resin, the initiator mainly changes the “mesh size”, while the plasticizer only affects “sponginess”, and they do so orthogonally to each other, as we present in Figure 10. The combination of these two topological parameters allows one to characterize any given network in an implicit but precise way and predict the resulting network properties, including the mechanical modulus. We believe that the same approach could also be useful for other polymer networks, including rubbers and gels.

## Abbreviations

The following abbreviations are used in this manuscript:

DPD	Dissipative particle dynamics
TCM	Three Chain Model
